# Theranostic application of *miR-429* in HER2+ breast cancer

**DOI:** 10.7150/thno.36274

**Published:** 2020-01-01

**Authors:** Claudia Cava, Chiara Novello, Cristina Martelli, Alessia Lo Dico, Luisa Ottobrini, Francesca Piccotti, Marta Truffi, Fabio Corsi, Gloria Bertoli, Isabella Castiglioni

**Affiliations:** 1 Institute of Molecular Bioimaging and Physiology, National Research Council (IBFM-CNR), Via F.Cervi 93, 20090 Segrate-Milan, Milan, Italy.; 2 Department of Pathophysiology and Transplantation, University of Milan, Milan, Italy.; 3 Laboratory of Nanomedicine and Molecular Imaging, Istituti Clinici Scientifici Maugeri IRCCS, via Maugeri 4, 27100, Pavia, Italy.; 4 Department of Biomedical and Clinical Sciences “L. Sacco”, Università degli studi di Milano, via G. B. Grassi 74, 20157 Milano, Italy.; 5 Breast Unit, Surgery Department, Istituti Clinici Scientifici Maugeri IRCCS, via Maugeri 4, 27100, Pavia, Italy.

**Keywords:** Theranostics, HER2+ breast cancer, microRNAs, diagnosis, therapeutic tool

## Abstract

Human epidermal growth factor receptor 2 (HER2) is overexpressed/amplified in one third of breast cancers (BCs), and is associated with the poorer prognosis and the higher metastatic potential in BC. Emerging evidences highlight the role of microRNAs (miRNAs) in the regulation of several cellular processes, including BC.

Methods: Here we identified, by *in silico* approach, a group of three miRNAs with central biological role (high degree centrality) in HER2+ BC. We validated their dysregulation in HER2+ BC and we analysed their functional role by *in vitro* approaches on selected cell lines and by *in vivo* experiments in an animal model.

Results: We found that their expression is dysregulated in both HER2+ BC cell lines and human samples. Focusing our study on the only upregulated miRNA, *miR-429*, we discovered that it acts as an oncogene and its upregulation is required for HER2+ cell proliferation. It controls the metastatic potential of HER2+ BC subtype by regulating migration and invasion of the cell.

Conclusions: In HER2+ BC oncogenic* miR-429* is able to regulate HIF1α pathway by directly targeting VHL mRNA, a molecule important for the degradation of HIF1α. The overexpression of *miR-429*, observed in HER2+ BC, causes increased proliferation and migration of the BC cells. More important, silencing *miR-429* succeeds in delaying tumor growth, thus *miR-429* could be proposed as a therapeutic probe in HER2+ BC tumors.

## Introduction

The St.Gallen International Breast Cancer Conference classifies breast cancer (BC) into five intrinsic molecular subtypes: luminal A, luminal B, HER2-enriched, basal-like, and normal-like 1. BC patients with overexpression of Human epidermal growth factor receptor 2 (HER2) have shown a poorer and shorter overall survival (OS) 2, 3 being HER2 expression a predictor of metastatic BC 4. Indeed, patients with HER2+ BC frequently develop metastases in the brain, liver and lung 5.

HER2 was discovered as a human proto-oncogene in 1985 through its homology with v-erbB and with the human epidermal growth factor receptor (EGFR) gene. HER2 gene encodes for a tyrosine kinase receptor, whose amplification and activation lead to uncontrolled cell proliferation. In 1987, D. Slamon described amplification of HER2 gene in ~30% of all clinical samples of BC 6. As a result, HER2 receptor is overexpressed in HER2+ BC patients up to 20 times more than in normal tissues 6. This overexpression is involved in the cell proliferation maintenance, avoiding apoptosis and in favoring cell spreading and metastasis formation 7.

The actual methods for HER2+ BC diagnosis are based on the analysis of tissue samples by immunohistochemistry (IHC) of HER2 protein or *in situ* hybridization of HER2 specific transcript. This method is widely used as it is cost-effective, easy to be used for rapid evaluation in primary diagnostics and for rapid screens 8. Although the optimization guides help the pathologists in the diagnosis 9, 10, the IHC approach is limited by technical features, such as the correct identification of the tumor areas within a complex tissue section, or the interpretation of the visual expression pattern and intensities in the tumor cells and in surrounding tissue 11. Poor reproducibility among different laboratories, or among observers and pathologists in the same laboratory has emerged (i.e. 12). Moreover, some studies highlight the existence of discordance in 25% of HER2+ BC cases between two different pathologists 13-15. These observations pushed the research towards the discovery of new reliable diagnostic biomarkers to be used in precision medicine for HER2+ diagnosis.

The main methods to define new robust biomarker are based on the analysis of high-throughput database, in order to identify differentially expressed genes, and on the integration of this information with functional pathways and with the biological relationships among cellular components (network) 16-19. These integrative approaches consider also epigenetic features of the tumors, with a particular attention to microRNA (miRNA) molecules. miRNAs are small non coding RNA with a main role in BC development, being able to regulate several mRNA targets. A small change in miRNA expression levels could have a high impact on the phenotype of the cell 20.

In our laboratory we developed a new *in silico* integrative approach to put all the information on differentially expressed genes, network and functional pathways in relation with miRNA molecules, in order to identify a specific group of miRNAs with a main role in the development of HER2+ BC. In particular, we identified three miRNAs, namely *Hsa-miR-190*, *Hsa-miR-429* and *Hsa-miR-584* (indicated from here on as *miR-190, miR-429* and* miR-584,* respectively), having a higher degree centrality in HER2+ BC, being able to control the highest number of genes in coupled functional pathways.

In this paper, we described the bioinformatics approach and validated the ability of these three miRNAs to perform differential diagnosis of HER2+ BC. Moreover, we focused on *miR-429*, the only upregulated miRNA of the three with a highest number of connections with the functional pathways, and we analyzed its role in controlling specific function altered in HER2+ BC. *miR-429* demonstrated to be a diagnostic molecule involved in the control of HIF1α pathway, having as a direct target VHL gene. Indeed, *in vitro* experiments revealed that *miR-429* is involved in the regulation of proliferation and metastatic potential of highly aggressive BC. In addition to its diagnostic properties, *in vivo* studies with xenografted mouse, showed that the treatment with a silencer oligonucleotide of *miR-429* is able to cause a delay of tumor proliferation, thus making *miR-429* a possible candidate molecule for the development of new therapeutic tools in HER2+ BC.

## Material and Methods

### Datasets for computational analysis

We applied the computational approach on HER2+ BC dataset of IlluminaHiSeq RNASeqV2 derived from The Cancer Genome Atlas (TCGA). We selected 43 HER2+ BC samples and 113 normal samples (NS). The expression levels of 1046 miRNAs and 15243 genes (excluding genes with a small variance) were considered. We used BC matched samples of mRNA and miRNA.

### Computational approach

The computational analysis consists of 4 steps. In the first step we applied a differential expression analysis identifying pathways enriched with differentially expressed genes between HER2+ BC samples and NS. In the second step we calculated a measure of pathway cross-talk among pairwise pathways. In the third step we selected pairwise pathways with a machine learning approach to distinguish HER2+ BC samples *vs* NS with the best performance, generating a pathway cross-talk network. In the fourth step we applied a mutual information analysis in order to identify differentially expressed miRNAs regulating pathway cross-talk.

**Figure 1:**
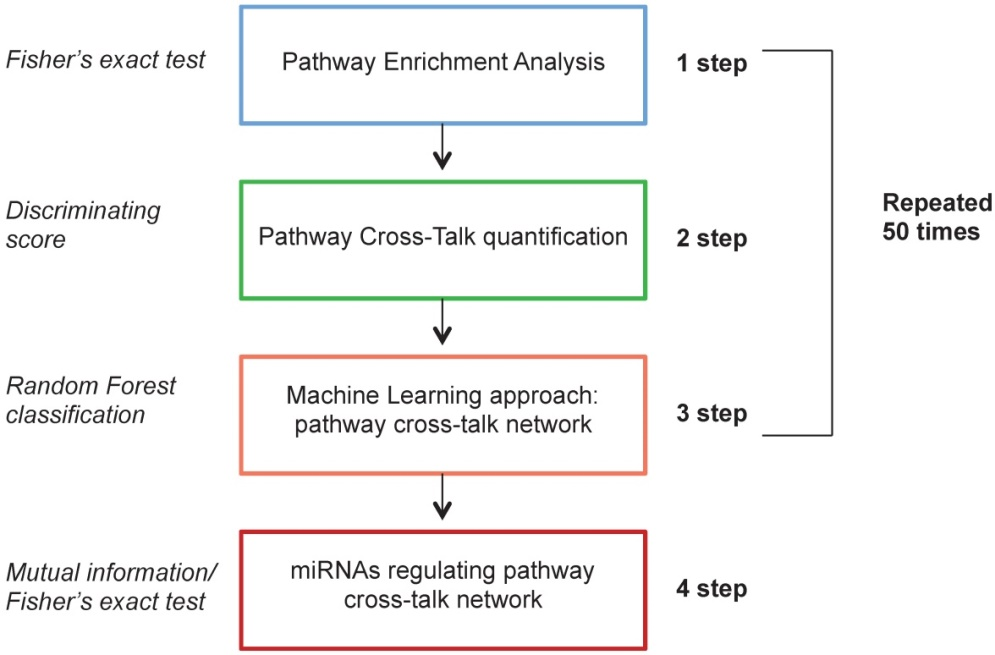
Workflow of the proposed computational approach.

The computational approach was based on a Monte Carlo Cross-Validation, dividing the original dataset into training (60%) and testing (40%) dataset. The first three steps were repeated 50 times on different generated training data set. The fourth step was applied on the whole dataset. To avoid problems of unbalanced classes, we generated randomly an equal number of samples for each class (HER2+ BC and NS). Figure 1 shows the workflow of the proposed computational approach.

### Pathway enrichment analysis

Differentially expressed genes between HER2+ BC and NS were identified by a statistical analysis using TCGAbiolinks package 21 (|logFC| >1 and *p*-value<0.01). The *p*-values were adjusted by the Benjamin-Hochberg method for multiple testing correction 22.

Considering 589 pathways from the Ingenuity Pathway Analysis (IPA) database, we carried out a pathway enrichment analysis. The enrichment was performed using the Fisher's Exact Test; we considered a pathway to be enriched with differentially expressed genes if the associated *p*-value was <0.01 23.

### Pathway cross-talk quantification

Interactions between enriched pathways were evaluated through a discriminating score (DS) 24. This score was calculated considering the mean and standard deviation of the gene expression levels for each couple of pathways. *M_x_* and *M_y_* represent the mean of gene expression levels for the pathway *x* and *y*; *SD_x_*and* SD_y_*are the standard deviation for the pathway *x* and *y*.

DS = 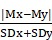


### Machine learning approach: pathway cross-talk network

We implemented a machine learning model using a Random Forest classification of the R-package 25(k-cross validation, k=10) in order to select the pairwise pathways that achieved the best performance in the classification of HER2+ BC and NS. The performances were valuated using the Area Under Curve (AUC). The classification was repeated 50 times for each different training dataset, selecting the top 10 pairs of pathways for each bootstrap. Finally, we selected the pathways that achieved the top 10 positions in a major number of boots. The AUC performances were validated also using the testing dataset.

### miRNAs regulating pathway cross-talk network

Differential expression analysis of miRNAs between HER2+ BC and NS was performed using TCGAbiolinks package (|logFC| >1 and adjusted *p*-value <0.01) 21.

Mutual information was applied between differentially expressed miRNAs in HER2+ BC *vs* NS and 15243 genes of the original mRNA dataset, using the R-package parmigene 26.

Since we obtained a list of genes potentially regulated by miRNAs, we evaluated the pathways enriched with target genes for each miRNA. In particular, using Fisher's exact test, we selected miRNAs that are able to regulate the network of pathways (*p*-values<0.01). Furthermore, we selected the miRNAs with the highest value of *degree centrality* in the network, namely those able to regulate the major number of genes in the pathways 27.

We generated a pathway cross talk network, considering only the pathways in the top 10 pairs of pathways that present at least 2 or more interactions with the other pathways and miRNAs.

### BC human tissue samples for *ex vivo* studies

We used ten samples of HER2+ BC human tissue and corresponding non-tumoral clear margins from surgical resections performed from 2011 to 2013 at the Breast Unit of Istituti Clinici Scientifici Maugeri IRCCS, Pavia, Italy. Samples referred to the biological collection of the “Bruno Boerci” Oncological Biobank for research applications (Istituti Clinici Scientifici Maugeri IRCCS, Pavia), a biobank certified to ISO 9001:2015 and member of BBMRI.it (the italian node of the BBMRI-ERIC, Biobanking and BioMolecular resources Research Infrastructure - European Research Infrastructure Consortium). Upon informed consent from patients, samples were collected, processed and stored at -80°C as snap-frozen aliquotes immediately after surgery, according to the best practices in biobanking (certification ISO 9001:2015). At the time of collection, HER2 enrichment was assessed through IHC molecular characterization by the Pathology Service (Istituti Clinici Scientifici Maugeri IRCCS, Pavia), according to the clinical guidelines on BC (ASCO - American Society of Clinical Oncology). Molecular characterization of the tumor samples is shown in Table S1. The samples were used for the isolation of total RNA and miRNA analysis in real-time PCR (as described in the methods below).

### BC cell culture for *in vitro* studies

We used SKBR3 cells (ICLC, Genova, Italy), that are the reference HER2+, human, BC epithelial cell line 28 and MCF10A cells, as human, normal-like epithelial breast cells 29. We maintained the cell line within a humidified atmosphere containing 5% CO_2_ at 37 °C in DMEM high glucose cell culture medium (Gibco, Life Technologies), with 10% fetal bovine serum (FBS), 1% penicillin/streptomycin, 2mM L-Glutamine (Euroclone) following the manufacturer's recommendation. Dulbecco Phosphate-Buffered Saline (D-PBS), and trypsin were obtained by Lonza (Euroclone).

### RNA extraction and real time-PCR analysis

Total RNA of both cell lines and human samples was isolated using TRIzol reagent (Life Technologies) following the manufacturer's recommendations and as already described in 29. For miRNA quantification, total RNA was reverse transcribed using MystiCq microRNA cDNA synthesis kit (Sigma Aldrich), following manufacturer's recommendations. miRNAs were amplified in Eco real time-PCR (RT-PCR) (Illumina, Euroclone) using Power Up Sybr green mix (Applied Biosystem) in combination with homemade designed primers (Sequences in the Table S2). For each RT-PCR analysis the results are presented as 2^-ΔΔCt method 30 normalizing the results on the expression of a *miR-103-3p* (Table S2) and the positive control of the MystiCq microRNA cDNA synthesis kit.

### miRNA modulation

miRNA increase or decrease were obtained in SKBR3 cell line by transfection of sense or antisense oligonucleotide (Sigma Aldrich) with metafectene reagents (Biontex, Germany), following manufacturer's instruction. The sequences for miRNA modulation are in Table S2. All miRNA oligonucleotides were designed on the sequences from miRbase database 31.

### MTT assay

SKBR3 cells were seeded in 96 wells at 5000 cells/well. 48h after seeding the cells were treated with Scramble S or As *miR-429* oligonucleotide for miRNA modulation. At 24, 48 and 72h after treatment, the cells were stained with 50ug/ul of thiazolyl blue tetrazolium bromide (MTT) (Sigma Aldrich) for 4 h at 37°C and then lysed in dimethyl sulfoxide (DMSO; Euroclone, Italy). The staining was quantified at 540nm in FLUOstar Omega microplate reader (Euroclone). The presented results are the average±standard deviation (sd) of three independent experiments.

### Wound-healing assay

The SKBR3 cells were seeded at a confluence of 100000/well in 24-well plate (Euroclone). After 48 h, necessary to reach 80% confluency, a scratch was performed in the middle of each well, as described in 29. After washing the cells, new fresh medium was added, and miRNAs were modulated by transfection, as described before. Pictures of each well were taken every 24 h. The effect on cell migration was quantified by ImageJ software, following the protocol described in 32. Experiments were performed in triplicate (*n* = 9) and a *t* test was calculated.

### Cell invasion in Boyden's chamber

To study the invasion ability of the cells, we performed the Boyden's chamber test on SKBR3 in the presence of *miR-429* silencing. Fifty thousand SKBR3 cells were seeded on the top of the 70ul coating Matrigel (BD) and covered with 350ul of complete medium except for 2% FBS. The lower chamber was filled with complete medium. One hour after seeding, the cells were treated with 150nM scramble or As *miR-429* oligonucleotides. Twenty-four hours later, cells migrated through the Boyden's chamber, were fixed in 4% PFA and stained with cresyl violet for 5 minutes (min.). Ten pictures for each condition were taken and the stained cells were count. The experiment was performed in triplicate and the average of the migrated cells is calculated.

### HER2+ BC animal model for *in vivo* treatment studies

Animal experiments were carried out in compliance with the institutional guidelines for the care and use of experimental animals (European Directive 2010/63/UE and the Italian law 26/2014), authorized by the Italian Ministry of Health and approved by the Animal Use and Care Committee of the University of Milan. Ten female nude mice (athymic nude-Foxn1 Nu/Nu, Envigo), 7-8 weeks old, were all xenografted to model HER2+ BC with 5 million SKBR3 cells in Matrigel injected in mammary gland. During *in vivo* study, mice were maintained on a 12-h light-dark cycle in cages of five animals with water and food ad libitum (environment of 23 ± 1°C and 50 ± 5% humidity). After detection of the tumors (about 20 days), the animals were divided in two groups: the first group was treated with 5 pM scramble S oligonucleotide in the presence of Atelogene as vehicle; the second group was treated with 5 pM As *miR-429* in the presence on Atelogene as vehicle. One animal was treated with vehicle alone. Tumor growth was followed over time by measurements of the three dimensions of the palpable masses (height x length x depth) and the tumor volume was calculated by multiplying the three dimensions of the tumor measured by caliper.

For *ex vivo* studies, at the end of the experiments, mice were sacrificed by cervical dislocation after sedation, and tumours were explanted for immune-histochemical and molecular analyses.

### Luciferase assay

To assess if *VHL* is a direct target of *miR*-*429*, pGL3-control vector or pGL3-VHL-WT was transfected with lipofectamine 3000 (Invitrogen) in SKBR3 cells, according to the manufacturer's suggestion, in combination with 100 nM mimic *miR*-*429* or *miR-142* (a known regulator of VHL) or scramble miR sequence. After 24 h, Gaussia Luciferase (GLuc) activity was measured in the collected medium and normalized on the Secreted Alkaline Phosphatase (SEAP) content. Results were detected by Glomax (GloMax-Multi Detection System; Promega, Madison, WI, USA).

### Western blot analysis

Western blot on total protein lysates was performed as described in 29. Anti VHL or anti actin antibodies (both Santa Cruz Biotechnologies) was used 1:500 overnight in 3% skimmed milk and the secondary anti-mouse HRP (Santa Cruz Biotechnologies) was used 1:1000 1 h at room temperature in 3% skimmed milk. Membranes were developed using the SuperSignal West Pico Chemiluminescent Substrate (ThermoScientific, France) on Amersham Hyperfilm MP (Ge Helthcare, Italy). The blots of both proteins were quantified by ImageJ software (http://imagej.nih.gov/ij/). Experiments were performed two times in triplicate.

### Hypoxia induction

In order to study the effects of hypoxia on *miR-429* expression, we used a hypoxic chamber 33. During hypoxia experiments, SKBR3 cells were seeded at 150000 cells/w in a 6 wells' plate and incubated in the hypoxic chamber containing 1% O_2_ gas mixture for 24 h (h). After this treatment, the cells were used for RNA extraction and RT-PCR analysis of *miR-429* expression.

## Results

### Computational approach predicted that *miR-190, miR-429 and miR-584* have a central role in the diagnosis of HER2+ BC

**Figure 2:**
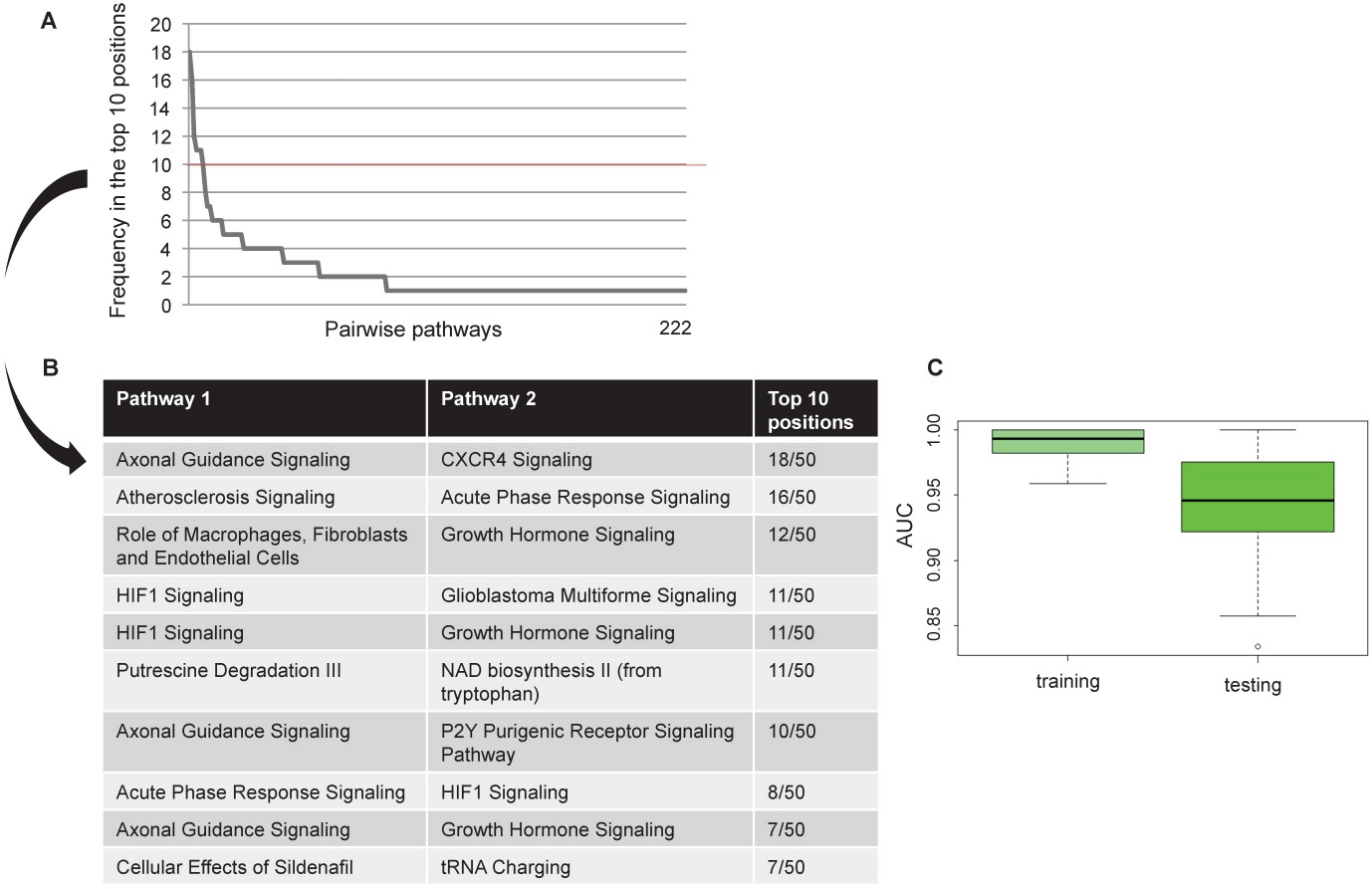
** Computational approach results** Frequency of pairwise pathways in the top 10 for all 50 bootstraps (A). Pathways achieving the best performance in all 50 bootstraps (B) Boxplot representation of the AUC values in the training and testing dataset (C).

We found 222 pairwise pathways that obtained an AUC value in the top 10 positions in at least one bootstrap in discriminating HER2+ BC versus NC. Figure 2A shows the distribution of the 222 pairwise pathways in the top 10 positions for each boot. Then, we selected the 10 pairwise pathways that achieved the best performances in all bootstraps (Figure 2B). Overall, the selected pairwise pathways obtained an AUC value of 0.98 in the training and 0.94 in the testing data set (Figure 2C).

We identified miRNAs regulating the selected pairwise pathways generating a diagnostic pathway-miRNA network.

**Figure 3:**
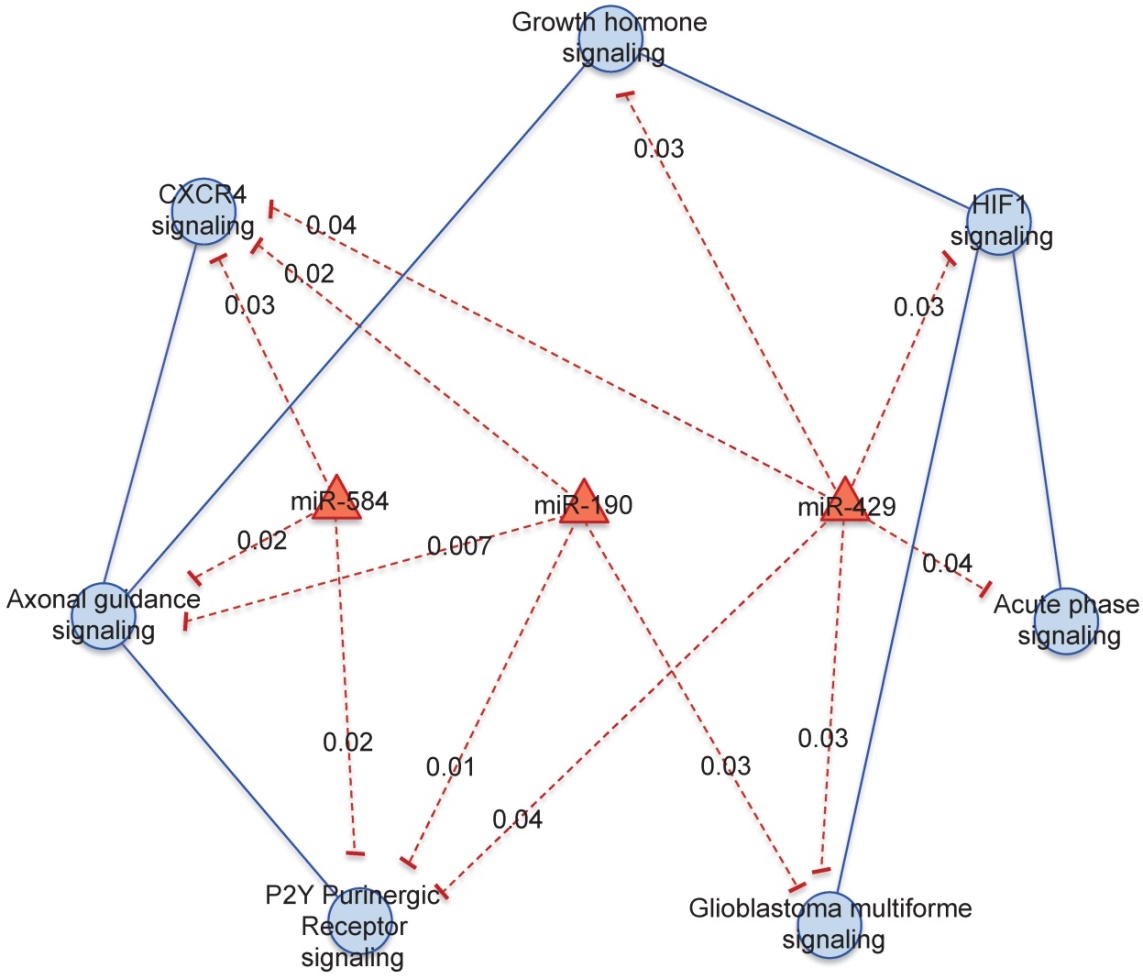
** Pathway cross-talk networks in HER2 BC and their regulatory miRNAs.** In red are indicated miRNAs and their connections, in blue are the functional pathways and their connections with the corresponding *p*-values.

We focused on 3 miRNAs altered in HER2+ BC indicating the number of genes potentially regulated by each miRNA obtained by mutual information approach, on the total number of genes in the coupled pathway. In our study we considered those miRNAs with the highest degree centrality, *miR-190*, *miR-429* and *miR-584*. From our analysis *miR-429* control 33 genes over 484 in 3 coupled pathways, *miR-190* and *miR-584* controls 30 and 49 genes over 693 in 3 and 2 coupled pathways, respectively (Table S3). We selected the pathways and miRNAs with the highest degree centrality (HDC) obtaining a network of 7 pathways and 3 miRNAs (Figure 3).

*miR-429* regulates the major number of pathways: "HIF1 signaling", "Acute Phase Response signaling", "Glioblastoma Multiforme signaling", "P2Y Purigenic Receptor signaling", "CXCR4 signaling " and “Growth hormone signaling”. *miR-584* regulates "P2Y Purigenic Receptor signaling", "CXCR4 signaling" and "Axonal Guidance signaling". *miR-190* regulates "P2Y Purigenic Receptor signaling", "Axonal Guidance signaling", "CXCR4 signaling" and "Glioblastoma Multiforme signaling".

### *In vitro* studies confirmed that miR-190, *miR-429 and miR-584* are diagnostic molecules for HER2+ BC

To evaluate if the expression levels of *miR-190*, *-429* and *-584* are different in HER2+ versus normal-like epithelial cells, as predicted by *in silico* analysis, we performed RT-PCR assay of these three miRNAs in both cell line and human BC samples. The SKBR3 HER2+ cells compared to MCF10A cells have significantly higher levels of *miR-429* compared to normal-like cells (Figure S1A and Figure S2) and lower levels of both *miR-190*, and *miR-584* (Figure S1B and C, respectively).

### *ex vivo* studies confirmed that *miR-190, miR-429* and *miR-584* are diagnostic molecules for HER2+ BC

**Figure 4:**
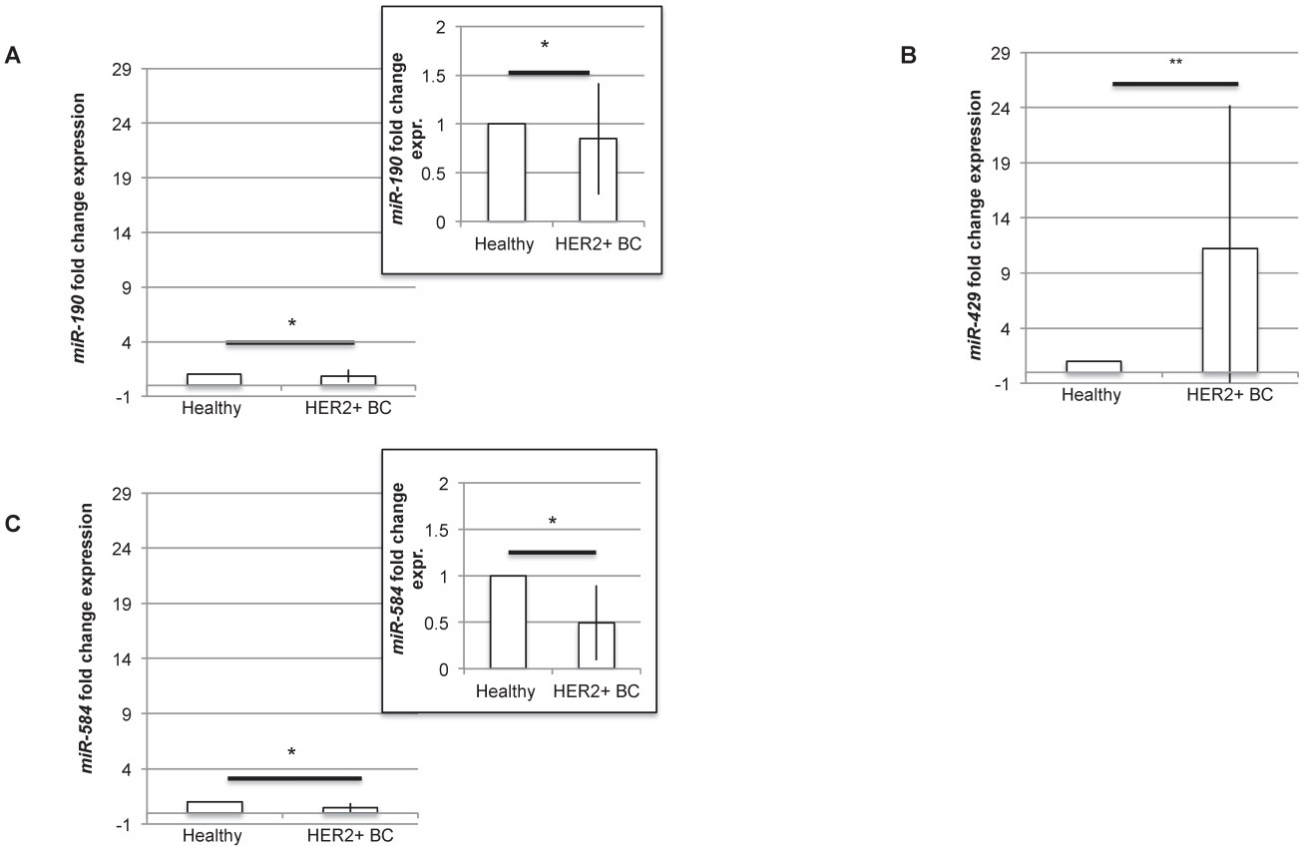
***miR-429* is overexpressed, while *miR-190* and *miR-584* are downregulated in HER2+ human BC.** RT-PCR analysis of the fold change expression levels of *miR-190* (A), *miR-429* (B) and *miR-584* (C) in HER2+ BC human samples compared to their corresponding healthy mammary tissue. The smaller panels in plot 4A and 4C represent an enlargement of the starting plot. The average value is indicated in red (n=10 samples, T test, p value<0.05, *).

In order to understand if the three miRNAs could be proposed as diagnostic molecules for HER2+ BC, we analysed the level of expression of *miR-190*, *miR-429* and *miR-584* in human BC HER2+ samples compared to normal surrounding epithelial mammary tissue. The RT-PCR experiment revealed that all the miRNAs are differentially expressed, being *miR-429* significantly upregulated and *miR-190* and* miR-584* significantly downregulated in human HER2+ BC samples (Figure 4A-C). In particular, considering the expression level of each miRNA in healthy tissue, *miR-190* and *miR-584* are downregulated to 0.84±0.57 and 0.49±0.40 respectively, while *miR-429* is upregulated to 11.23±12.95.

### *In vitro* downregulation of miR-429 blocked the proliferation and decreased migration and invasion of HER2+ BC cells

**Figure 5:**
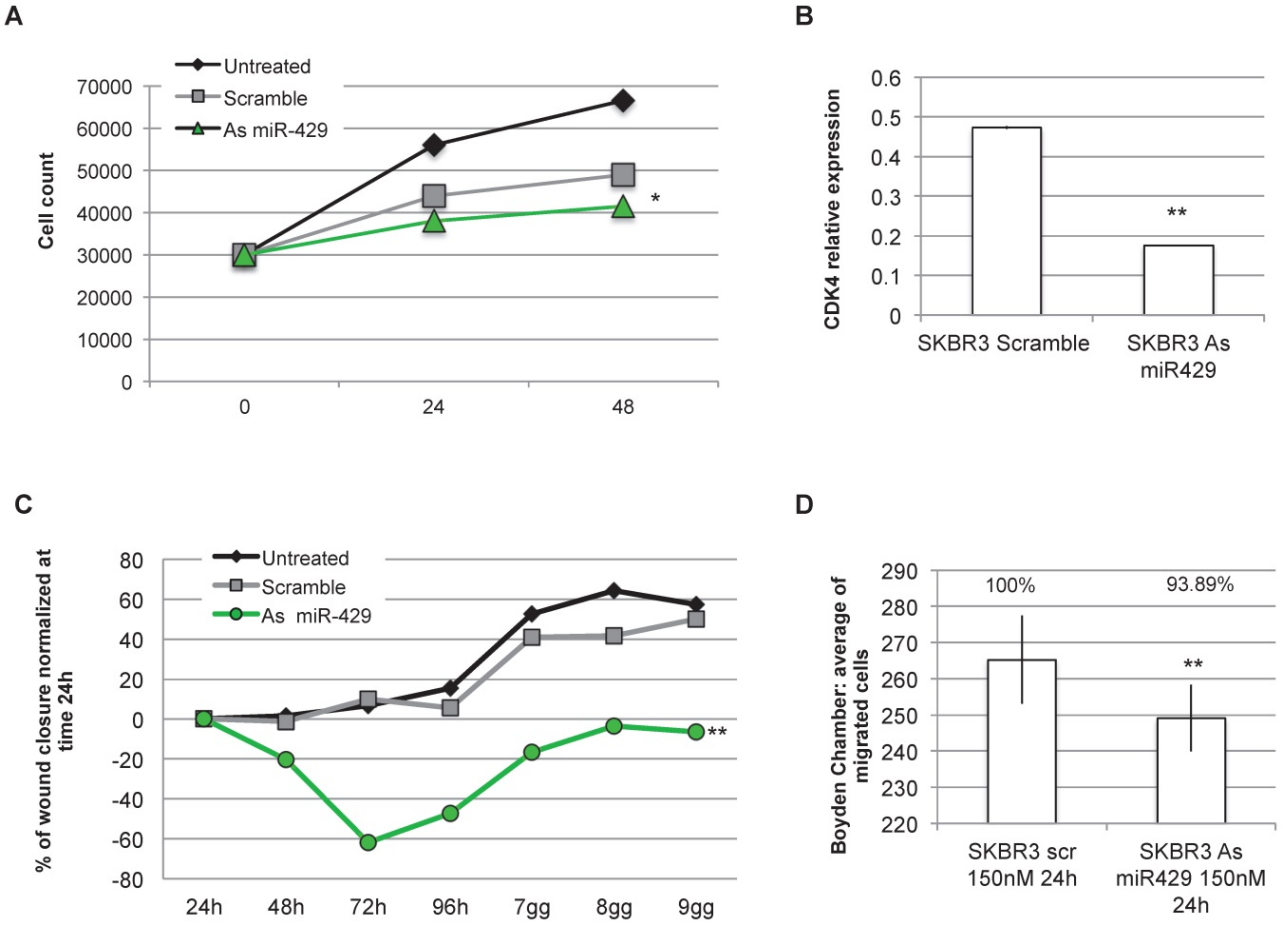
***miR-429* controls HER2+ BC cell proliferation , migration and invasion**. MTT assay was performed on SKBR-3 cell at 0, 24, 48 h after 200nM Scramble or As *miR-429* oligonucleotide treatment. The results are the average of three independent experiments in triplicate (t test compared to scramble treated cells, p value <0.05, *; <0.01, **)(A). 200nM 72h treatment with As *miR-429* reduced the expression of CDK4 significantly (t test n=2 experiments in triplicate, p value <0.01**)(B). Wound healing test was performed on SKBR-3 treated with 200nM scramble oligonucleotide or As *miR-429* in culture for 9 days. In each sample, the wound area was quantified by Image J following 31(t test compared to scramble-treated cells; n=3 experiments in triplicate, p value <0.01, **) (C). Boyden's chamber test was performed on SKBR-3 in the presence of 150nM scramble or As *miR-429* in culture for 24h. The count of the cells was performed on 10 images from each sample (t test compared to scramble-treated cells; n=3 experiments, p value<0.01, **) (D).

As *miR-429* role in HER2+ BC is unknown and as it is the only miRNA upregulated in HER2+ BC, we analysed the effect of *miR-429* silencing in HER2+ BC cell line. No correlation was observed between the different stages of BC and the *miR-429* expression level (Figure S3). Several of the target genes of *miR-429* are involved in the control of proliferation (i.e. 'HIF1 signaling'). We thus analyzed the effect of miRNA reduction on proliferation, by MTT assay. The significant downregulation of *miR-429*, obtained by 200nM As oligonucleotide treatment in SKBR3 cells for 72 h (Figure S4A), caused a delay in the proliferation rate (Figure 5A), that was statistically significant compared to scramble-treated cells (*t test* p value <0.05 *, <0.01 **). Similar results were obtained on another HER2+ BC cell line (MDA-MB-453, data in Figure S4C).

The observed reduction of proliferation rate observed in SKBR3 cells treated with As *miR-429* could be due to a reduction of CDK4 expression, thus possibly causing a block in G1 to S phase transition (Figure 5B).

Considering that *miR-429* is predicted by *in silico* analysis to regulate also pathways involved in cell migration and invasion (i.e CXCR4 pathway), we focused on the effects of As *miR-429* modulation in wound healing and Boyden's chamber tests. In the first assay, we quantify the area of the wound at each time point in SKBR3 cells treated with Scramble S or As *miR-429* oligonucleotides and normalized on that of the 24h (as suggested in 32) (Figure S4B), calculating the percentage of wound closure at each time point. The delay of the As *miR-429* treated SKBR3 cells in wound closure was significantly different from the scramble-treated cells (Figure 5C).

In the second assay, we observed that the treatment of 150nM As *miR-429* reduced significantly the migration ability of HER2+ SKBR3 cells compared to the scramble-treated cells (Figure 5D).

### *In vivo* downregulation of *miR-429* blocked tumor proliferation

**Figure 6:**
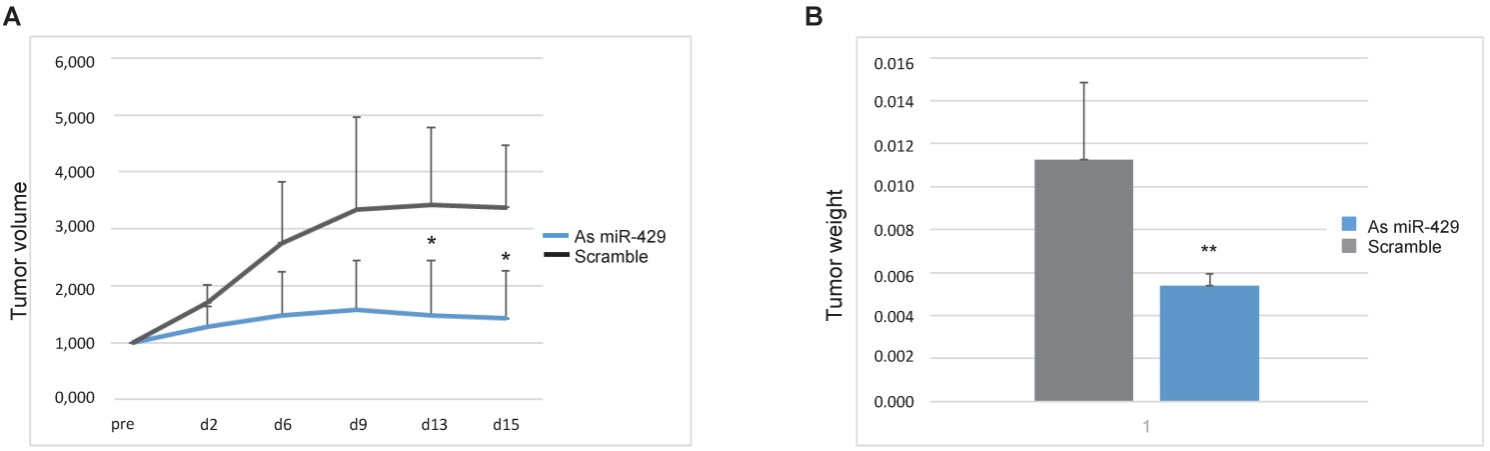
** As *miR-429* counteracts the growth of HER2+ BC tumors xenografted in mouse.** Tumors formed by SKBR3 in NOD/SCID mouse were treated with As *miR-429* or scramble S oligonucleotide in the presence of atelogene reagent (vehicle). The volume of each tumor (height x length x depth) was measured at different time points and the curves represent the average volume in 15 days of measurements (A). The weights of the five Scramble S- or As *miR-429*-treated tumors are represented (t test p value<0.01, **, n=5) (B).

To propose *miR-429* as a target for therapeutic tool development for HER2+ BC, we tested the efficacy of As *miR-429* treatment on the *in vivo* growth of HER2+ BC tumors. In particular, we treated the tumors formed by 5 million SKBR3 xenografted in ten NOD/SCID mouse mammary glands with different condition (Figure S5A). The animals treated with atelogene vehicle (n=1), or scramble oligonucleotide and atelogene (n=4) developed rapidly growing tumors. In those treated with As *miR-429* and atelogene (n=5) the growth of the tumor was delayed, with a significant difference at day 13 and 15 after therapeutic treatment, measured by tumor volume (Figure 6A), and by tumor weight at day 15 (Figure 6B), when the tumors were explanted and the mice were sacrificed. Representative pictures of treated tumors are presented in Figure S5B. In these tumors, we observed that the reduction of *miR-429*, obtained by As treatment, induced VHL increase and a parallel decrease of HIF1α targets, such as SLUG, VEGF and SNAIL (Figure S7).

### VHL signaling pathway is target of *miR-429*

**Figure 7:**
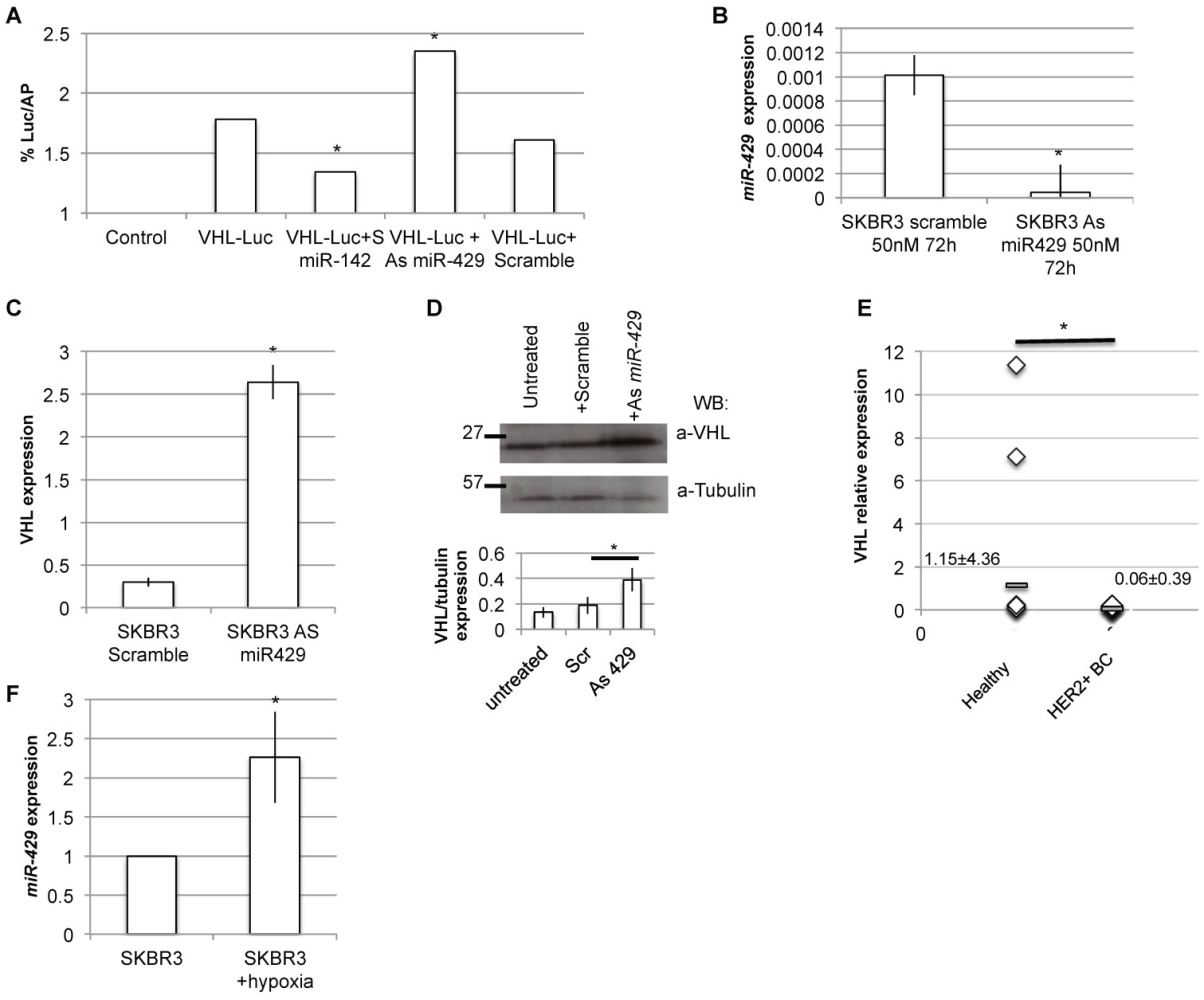
***VHL* is the direct target of* miR-429*.** Luciferase assay: HER2+ cells were transfected with the Luciferase+ 3'UTR VHL (pGL3-VHL) construct alone, with 100nM 48h S Scramble or S *miR-142* or As *miR-429*. The luciferase expression (LUC) was normalized on alkaline phosphatase content (AP). The chart represents the percentage of LUC/AP ratio (T test, p value<0.05, *; n=2 independent experiment)(A). RT-PCR analysis revealed that *miR-429* modulation, obtained with 50nM As *miR-429* for 72h (B), caused a significant increase of VHL expression, both at mRNA (C) and protein levels (D) (T test, p value<0.05, *; n=3 independent experiment). RT-PCR analysis on human HER2+ BC samples revealed that *miR-429* is downregulated, compared to healthy tissue (E).

As VHL is predicted to be a putative *miR-429* direct target (TargetScan and miRbase bioinformatics tools), we analyzed in detail the 3' untranslated regions (3'UTR) of human VHL target mRNA, and we found a possible seed for *miR-429* (Figure S6). Thus, the dual luciferase reporter assay was performed to test if *miR-429* is able to directly interact with 3'UTR of VHL. When we overexpressed the luciferase-3'UTR VHL construct in the presence of As *miR-429*, we observed a significant increase in luciferase activity compared to that observed in the cells co-trasfected with the same construct and the scramble oligonucleotide (Figure 7A). As a control, we performed the same transfection using the S *miR-142*, a known regulator of 3'UTR of human VHL 34.

Moreover, the *in vitro* downregulation of *miR-429* in HER2+ SKBR3 (Figure 7B) cells also caused a parallel upregulation of VHL mRNA and protein expression (Figure 7C and D, respectively).

To confirm the possible relation among *miR-429* and VHL target, we performed RT-PCR analysis of VHL expression on the human BC samples: as expected, VHL is downregulated in human HER2+ BC samples, compared to healthy surrounding tissue (Figure 7E).

### miR-429 is induced by hypoxia

**Figure 8:**
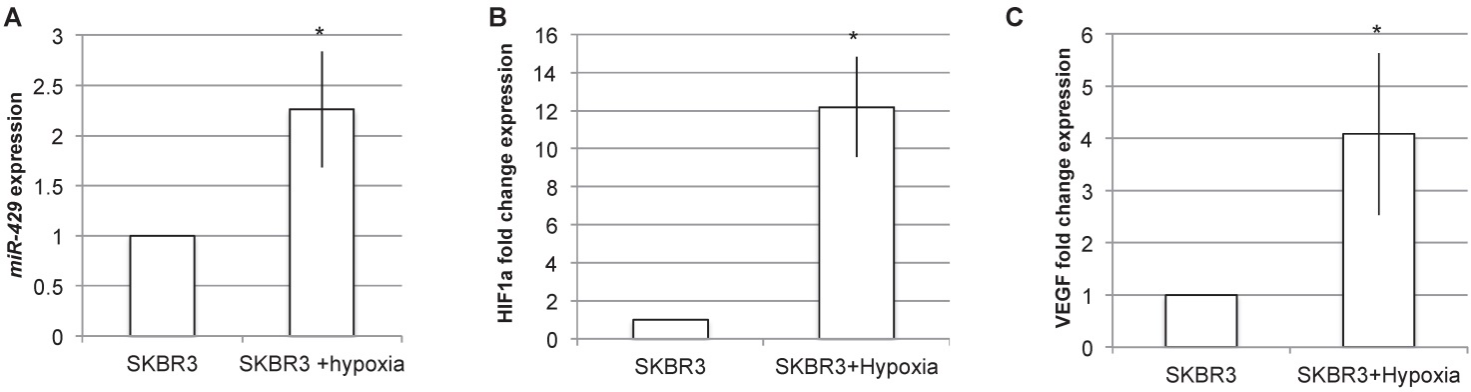
***miR-429* is induced by hypoxia.** RT-PCR analysis on SKBR3 revealed that 24h of hypoxia is able to induce *miR-429* increased expression (A). The hypoxia induction has been verified by the RT-PCR analysis of HIF1α and its target VEGF mRNA induction (B).

Being VHL a key component of hypoxia pathway, we analyzed the effect of hypoxia induction on *miR-429* expression levels (Figure 8). The RT-PCR analysis of *miR-429* expression in SKR3 after 24h of hypoxia treatment revealed that this condition is able to statistically increase *miR-429* expression (Figure 8A). The efficacy of hypoxia induction was evaluated by analyzing HIF1α and its target VEGF mRNA expression, which are upregulated after hypoxia treatment (Figure 8B and C).

## Discussion

Different miRNA signatures have been already proposed to have diagnostic or prognostic properties (i.e.^35^, ^36^). In this paper, we evaluated the diagnostic potential of three miRNAs, *miR-190*, *miR-429* and *miR-584*, and the theranostic role of *miR-429* in HER2+ BC. Although these miRNAs could be de-regulated in other BC subtypes, only in HER2+ they are altered being regulators of a specific pathway cross-talk network. Indeed, these miRNAs have been selected with an integrative bioinformatics approach relying on those miRNAs with a higher degree centrality in controlling a group of functional pathways containing differentially expressed genes, altered in HER2+ BC *versus* normal mammary tissues. RT-PCR analysis confirmed that *miR-429*, as well as *miR-190* and *miR-584*, are differentially expressed in both HER2+ BC cell lines, and in human HER2+ BC tissues (*miR-190* and *miR-584* downregulated and *miR-429* upregulated). Although *miR-584* and *miR-190* are already known tumor suppressor (i.e. 37, 38), our results suggested a possible use of all these 3 miRNAs for diagnostic purpose.

We selected *miR-429* as a possible therapeutic molecule, being the only upregulated miRNA among the three, and having a high degree centrality in controlling differentially expressed genes involved in "HIF1 signaling", "Acute Phase Response signaling", "Glioblastoma Multiforme signaling", "P2Y Purigenic Receptor signaling", "CXCR4 signaling " and “Growth hormone signaling”. In other tumors, as colorectal cancer, non-small cell lung cancer and triple negative BC, *miR-429* has been proposed as a regulator of metastatic properties of the cells 39, 40. Our *in vitro* experiments on HER+ cells suggested that *miR-429* has a role in controlling cell proliferation (MTT assay), possibly via Cyclin-dependent kinase 4 (CDK4) as in colorectal cancer 41, and confirmed also that *miR-429* is involved in migration and invasion (wound healing and Boyden's chamber tests, respectively). These results, obtained by As *miR-429*, could be due to its direct targeting of *VHL* gene (dual luciferase reporter assay). *VHL* codifies for a tumor suppressor protein involved in the degradation of hypoxia-inducible factor alpha (HIF1α) and the stromal-derived factor-1 receptor (CXCR4) proteins, which leads to the block of angiogenesis and tumor cell migration 42. It is already known that VHL is underexpressed in highly aggressive and grade 2-grade 3 BC tumors, with a specific significant reduction of VHL expression in those tumors that form recurrence 43. As VHL is a component of the HIF1α pathway, as hypoxia is one of the main functions affected in solid tumors (i.e. 44), and having VHL increase an impact on triple negative BC cell growth 45, we speculated that the As *miR-429* treatment, increasing mRNA of VHL, impacts on HIF1α protein degradation, leading to cell proliferation inhibition and to the block of metastatic potential of the HER2+ cells. Our *in vitro* experiments proved that As *miR-429* caused a delay of HER2+ cell growth and block invasion and migration ability of the cells. Our* in vivo* experiments on HER2+ BC mouse models confirmed that the silencing of *miR-429* impact on tumor cell growth and on tumor volume. These effects on cell proliferation and cell migration/invasion are possibly due to the decrease of HIF1α target mRNAs, such as SNAIL, VEGF and SLUG, (as shown in Figure S5) suggesting the use of As *miR-429* as a new therapeutic option for HER2+ BC patients. Moreover, we showed that the hypoxia treatment, which is a common state for several HER2+ BC tumors, induces *miR-429* expression; indeed, it has been demonstrated that overexpression of HER2 is able to induce HIF1 pathway components 46. Further research is needed to understand the *in vivo* effects of As *miR-429* on metastatic potential of HER2+ BC tumors. However, the use of As *miR-429* could be considered as a novel tool to be combined with classical chemotherapeutic drugs, such as trastuzumab, for HER2+ BC treatment. Some limitations of the use of miRNA-based therapy need to be overcome, in particular those regarding the systemic toxicity and the tissue targeting of the miRNA 47. Nevertheless, the combination of two drugs, one miRNA-based and one chemotherapeutic agent, could increase benefits to the patient by reducing the dose of chemotherapy and thus lowering its systemic toxicity.

In order to reduce the miRNA-mediated off-target effects and to better address the miRNA-based therapy to HER2+ BC tissues, we could take adantage of new emerging approach, i.e. those that use lipo-complexes conjugated with tumor targeting molecules, cationic polymers, conjugation of miRNA with lipids or receptor-binding molecules to increase cellular uptake, limiting the spreading of the drug in other organs 48.

In conclusion, from our results we proposed *miR-190*, *miR-429* and *miR-584* as diagnostic molecules for HER2+ BC, being *miR-190* and *miR-584* downregulated and *miR-429* upregulated in HER2+ BC. The *miR-429* increase could be induced by hypoxia, a common phenomenon for HER2+ BC. The silencing of *miR-429*, by regulating cell proliferation and invasion by targeting VHL mRNA, could be proposed a new anti-proliferative and anti-metastatic therapeutic strategy, alone or in combination with other drugs, for HER2+ BC patients' treatment.

## Supplementary Material

Supplementary figures and tables.
