# Supplementary material for: Theranostic application of miR-429 in HER2+ breast cancer
Source: Theranostics. 2020 Jan 1;10(1):50–61. doi: 10.7150/thno.36274 (PMC6929607; doi:10.7150/thno.36274)

Supplementary Material

Legends

Table S1. IHC characterization of the HER2+ BC subtype. Expression of estrogen receptor (ER), progesterone receptors (Pr), Ki-67 and C-erbB-2 in breast cancer tumor tissues.

Table S2
Sequences of the primers for RT-PCR miRNA amplification (in white) or miRNA modulation (in gray).

Table S3 *miR-429, miR-190 and miR-584* putative targets in HER2+ BC samples. For each miRNA (1st column) we indicated the pairwise pathways (2nd column), the number (3rd column) and the name of the target genes (4-5th column) within these pathways.

Figure S1 *miRNA-429* is upregulated, while *miR-190* and *miR-584* are downregulated in HER2+ BC cell lines. The expression levels of *miR-429* (A), *miR-190-5p* (B) and *miR-584* (C) were evaluated in SKBR3 HER2+ cell line compared to MCF10A 'normal-like' cell line, by RT-PCR analysis. The results are the average of three independent experiments in triplicate (t test, p value <0.05, *; <0.01, **, <0.001, ***).

Figure S2 RT-PCR analysis revealed that miR-429 is specific for HER2+ BC. RT-PCR analysis of *miR-429* expression level in Luminal A, Luminal B and HER2+ BC compared to normal-like cell lines. The results are the average of three independent experiments in triplicate. T test, p value<0.05,*; <0.01, **; <0.001, ***.

Figure S3. miR-429 is significantly overexpressed in each of the 3 considered stages compared to normal samples (t test p value<0.001, ***) (3A). Each stage composition is variable, containing either Luminal A Luminal B, Her2+ and basal. We observed that miR-429 expression is not associated with different stages of BC.

Figure S4 *miRNA-429* controls HER2+ BC cell proliferation migration and invasion.
In order to verify if miR-429 is involve in cell proliferation, the SKBR-3 cells were treated with 200nM of As *miR-429*. The expression level of *miR-429* was evaluated by RT-PCR analysis (t test compared to scramble treated cells, p value <0.01, **; <0.001, ***) (A). Wound healing test was performed on SKBR-3 treated with 200nM scramble oligonucleotide or As *miR-429* in culture for 9 days. In each sample, the wound area was quantified by Image J analysis and normalized on the same area at time 0 [31] (t test compared to scramble-treated cells; n=3 experiments in triplicate, p value <0.01, **) (B).
Cell count was performed on HER2+ MDA-MB-453 cell line at 0, 24, 48, 72, 96 hours of 200nM scramble or As *miR-429* oligonucleotide treatment. The results are the average of three independent experiments in triplicate (t test performed on scramble treated cells, p value<0.05, *)(C).

Figure S5 As *miR-429* is counteracts the growth of HER2+ BC tumors xenografted in mouse.
Schematic representation of tumor injection and As *miR-429* treatment workflow (A). The pictures represent the tumors, treated with As *miR-429* (on the left) or with scramble oligonucleotides (on the right) after explanting from the mouse (n=5 tumors from SKBR3 cells, T test, p value<0.05, *; p value <0.01, **) (B).

Figure S6 Analysis of the mRNA sequence of human VHL revealed a seed for *miR-429* pairing in the 3' UTR of the target.

Figure S7 RT-PCR analysis on tumors formed by scramble- or As *miR-429* treated SKBR3 cells, explanted from mouse. The tumors, in which we succeeded in *miR-429* reduction (A), have a higher VHL expression (B), reduction in HIF1α-target SLUG (C), VEGF (D) and SNAIL (E) expression in respect to Scramble-treated tumors. T test, p value <0.05, *; < 0.01, **.

Table S1.

#sample	% ER	% Pr	%Ki67	C-erbB-2
1	0	0	30	2+
2	0	0	15	3+
3	0	0	15	3+
4	0	0	15	3+
5	0	0	30	3+
6	0	0	15	3+
7	0	0	30	3+
8	0	0	15	3+
9	0	0	20	3+
10	0	0	15	3+
11	0	0	20	3+

Table S2
Sequences of the primers for RT-PCR miRNA amplification (in white) or miRNA modulation (in gray)

miRNA ID	Sequence (5'-3')
miR-429 (MIMAT0001536*)*	TCTAATACTGTCTGGTAAAACCGT
*miR-190a-5p (*MIMAT0000458)	GTGTGATATGTTTGATATATTAGGTTGTT
miR-584-5p (MIMAT0003249)	TTATGGTTTGCCTGGGACTGAG
miR-103-3p	AGCAGCATTGTACAGGGCTATGA
Scramble Sense (S)	ATGATGTCCTTCTAGTACGCATC
miR-429 AntiSense (As)	ACGGTTTTACCAGACAGTATTA

Table S3

miRNA	Pairwise pathways	n. of genes/tot genes	Genes in pathway A	Genes in pathway B
Hsa-miR-429	A) Acute phase response signaling;<br>B) HIF1 signaling	33/484	*CP, IL18, MAP2K7, NRAS, SERPINA1, SERPING1*	*ATM, CREBBP, NRAS, PIK3C3, VEGFB*
	A) HIF1 signaling<br>B) Glioblastoma multiforme signaling		*ATM, CREBBP, NRAS, PIK3C3, VEGFB*	*ATM, CDK2, E2F5, NRAS, PIK3C3, RHOC*
	A) HIF1 signaling<br>B) Growth hormone signaling		*ATM, CREBBP, NRAS, PIK3C3, VEGFB*	ATM, PIK3C3, PRKCB, RPS6KA2, SOCS6
Hsa-miR-190	A) Axonal guidance signaling<br>B) CXCR4 Signaling	30/693	*ADAM8, BMP1, FZD9, GNB2L1, HRAS, NGEF, PIK3C3, PIK3CB, ROCK1, SEMA3E*	*GNB2L1,HRAS, PIK3C3, PIK3CB, ROCK1*
	A) Axonal guidance signaling<br>B) P2Y Purinergic Receptor signaling pathway		*ADAM8, BMP1, FZD9, GNB2L1, HRAS, NGEF, PIK3C3, PIK3CB, ROCK1, SEMA3E*	*GNB2L1, HRAS, NFKB1, PIK3C37, PIK3CB*
	A) HIF1 signaling<br>B) Glioblastoma multiforme signaling		*COPS5, CREBBP, MMP15, MMP24, NOS3, NRAS, PIK3CA*	*FOXO1, FZD8, NRAS, PDGFRB, PIK3CA, PLCL1, RHOH, RHOQ*
Hsa-miR-584	A) Axonal guidance signaling<br>B) CXCR4 Signaling	49/693	*ARHGEF12, ARPC3, ARHGEF15, ATM, FARP2, GNAI1, GNB2, MAP2K1, NRAS, PLCB2, PLCD3, PXN, TUBA4A, UNC5B, WAS, WIPF1, WNT10A*	*ATM, GNAI2, GNB2, MAP2K1, MAPK12, NRAS, PLCB2, PXN*
	A) Axonal guidance signaling<br>B) P2Y Purinergic Receptor signaling pathway		*ARHGEF12, ARPC3, ARHGEF15, ATM, FARP2, GNAI1, GNB2, MAP2K1, NRAS, PLCB2, PLCD3, PXN, TUBA4A, UNC5B, WAS, WIPF1, WNT10A*	*ATM, GNAI2, GNB2, MAP2K1, NRAS, PLCB2, PLCD3*

Figure S1 *miRNA-429* is upregulated, while *miR-190* and *miR-584* are downregulated in HER2+ BC cell lines.
The expression levels of *miR-429* (A), *miR-190-5p* (B) and *miR-584* (C) were evaluated in SKBR3 HER2+ cell line compared to MCF10A ‘normal-like’ cell line, by RT-PCR analysis. The results are the average of three independent experiments in triplicate (t test, p value <0.05, *; <0.01, **, <0.001, ***).

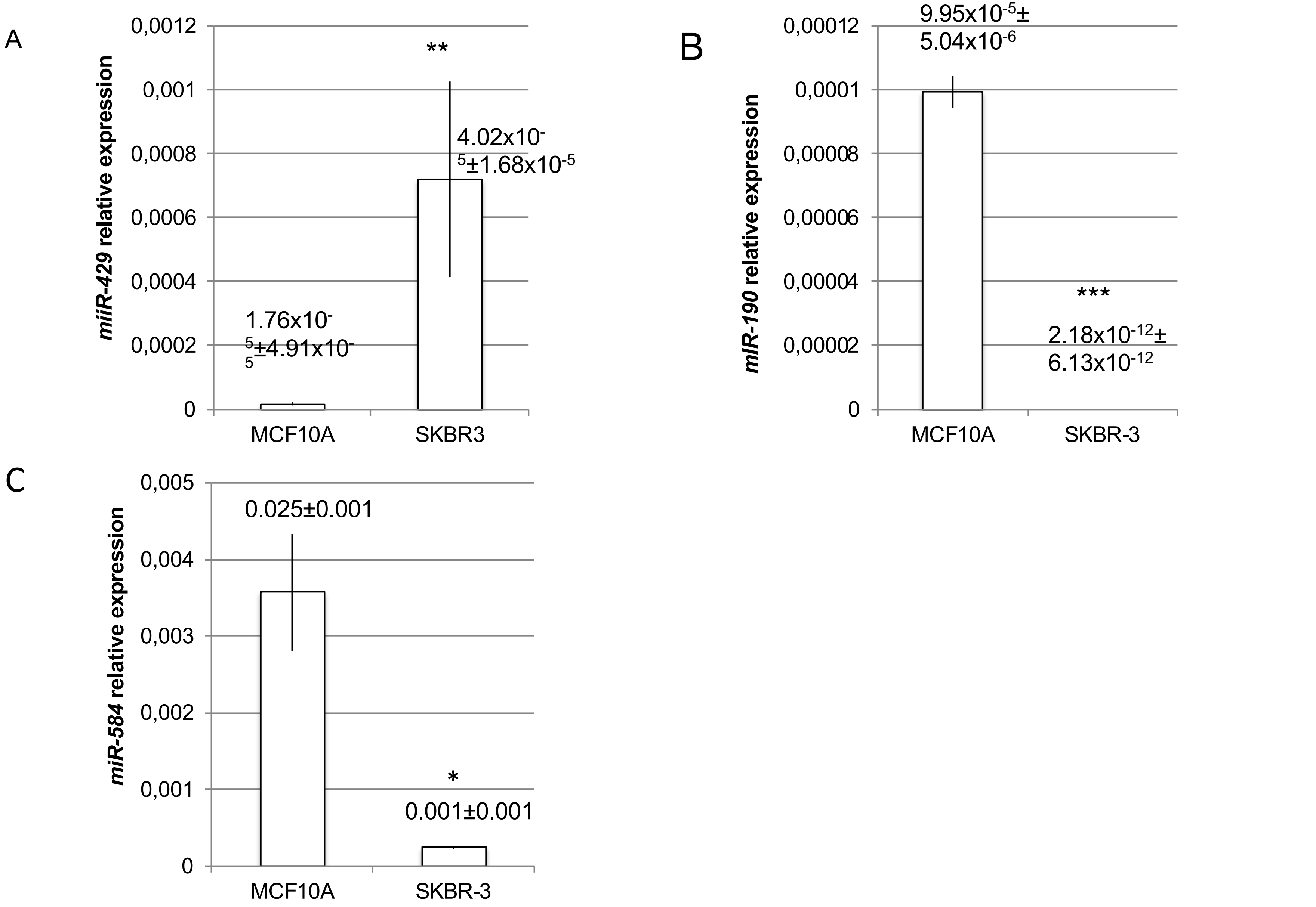

Figure S2 RT-PCR analysis revealed that miR-429 is specific for HER2+ BC. RT-PCR analysis of miR-429 expression level in Luminal A, Luminal B and HER2+ BC compared to normal-like cell lines. The results are the average of three independent experiments in triplicate. T test, p value<0.05,*; <0.01, **; <0.001, ***.

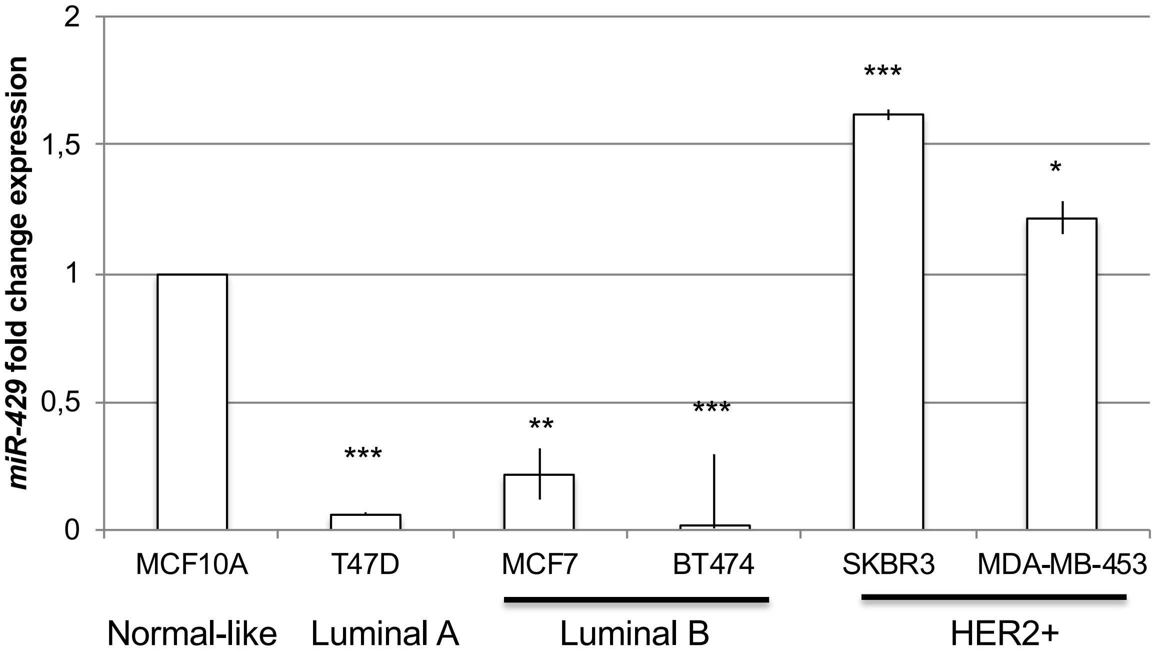

A

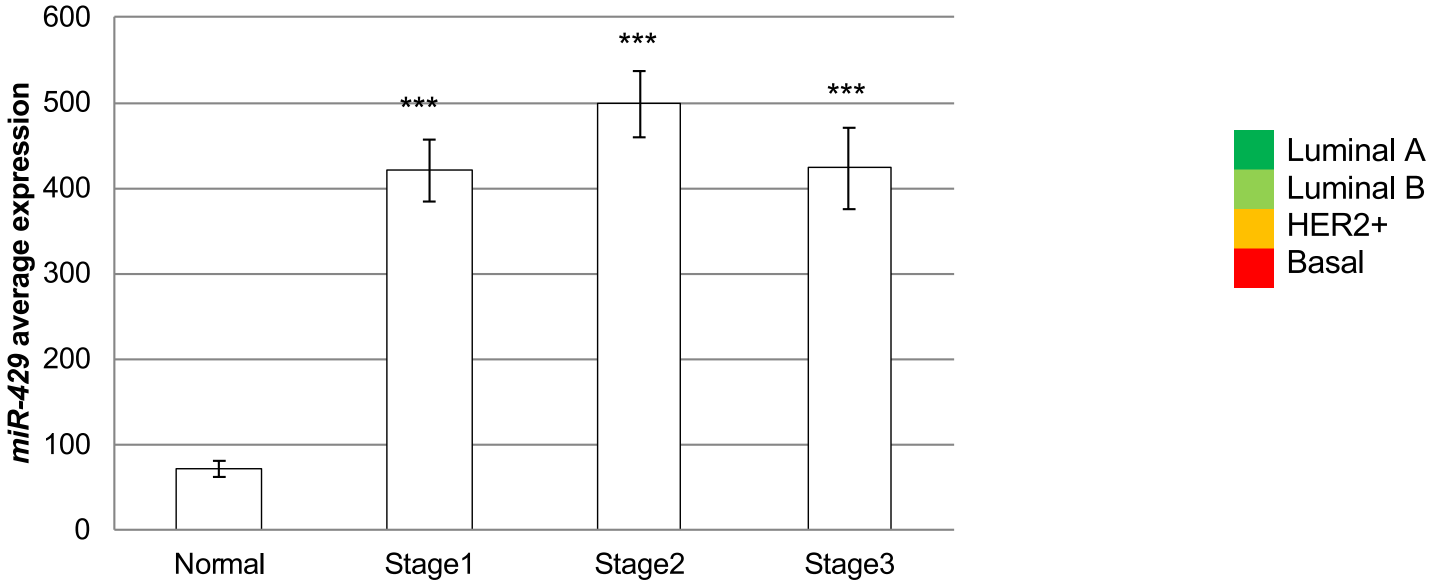

B

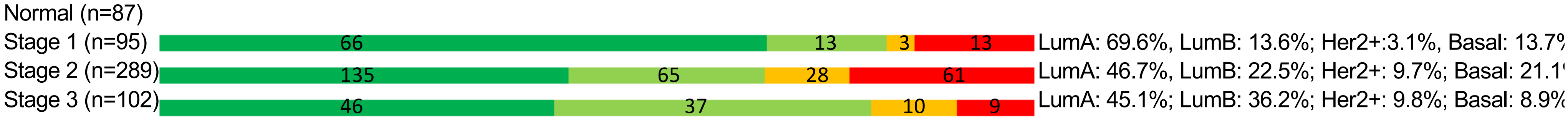

LumA: 247 total
LumB: 115 total
Her2+: 41 total
Basal: 83 total

Figure S3: *miR-429* is significantly overexpressed in each of the 3 considered stages compared to normal samples (t test p value<0.001, ***) (3A). Each stage composition is variable, containing either Luminal A Luminal B, Her2+ and basal. We observed that *miR-429* expression is not associated with different stages of BC.

Figure S4 *miRNA-429* controls HER2+ BC cell proliferation blocking G1/S transition.
The SKBR-3 cells were treated with 200nM of As *miR-429*. The expression level of *miR-429* was evaluated by RT-PCR analysis (t test compared to scramble treated cells, p value <0.01, **; <0.001, ***) (A).
Wound healing test was performed on SKBR-3 treated with 200nM scramble oligonucleotide or As *miR-429* in culture for 9 days. In each sample, the wound area was quantified by Image J analysis and normalized on the same area at time 0 (t test compared to scramble-treated cells; n=3 experiments in triplicate, p value <0.01, **) (B).
Cell count was performed on HER2+ MDA-MB-453 cell line at 0, 24, 48, 72, 96 hours of 200nM scramble or As miR-420 oligonucleotide treatment. The results are the average of three independent experiments in triplicate(t test performed on scramble treated cells, p value<0.05, *)(C).

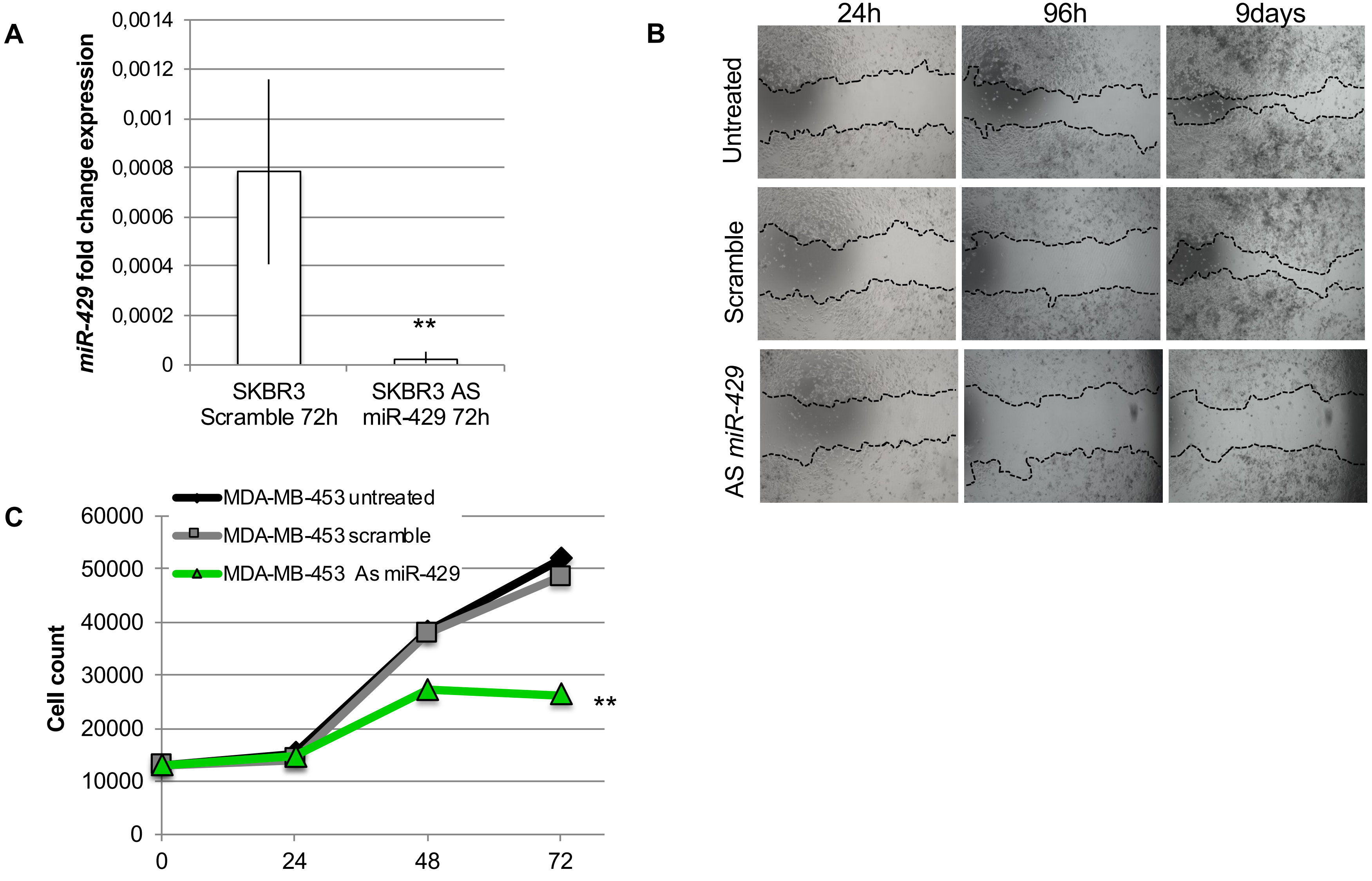

Figure S5

(A) Schematic representation of tumor injection and As *miR-429* treatment workflow

(B) The pictures represent the tumors, treated with As *miR-429* (on the left) or with scramble oligonucleotides (on the right) after explanting from the mouse (n=5 tumors from SKBR3 cells, T test, p value<0.05, *; p value <0.01, **).

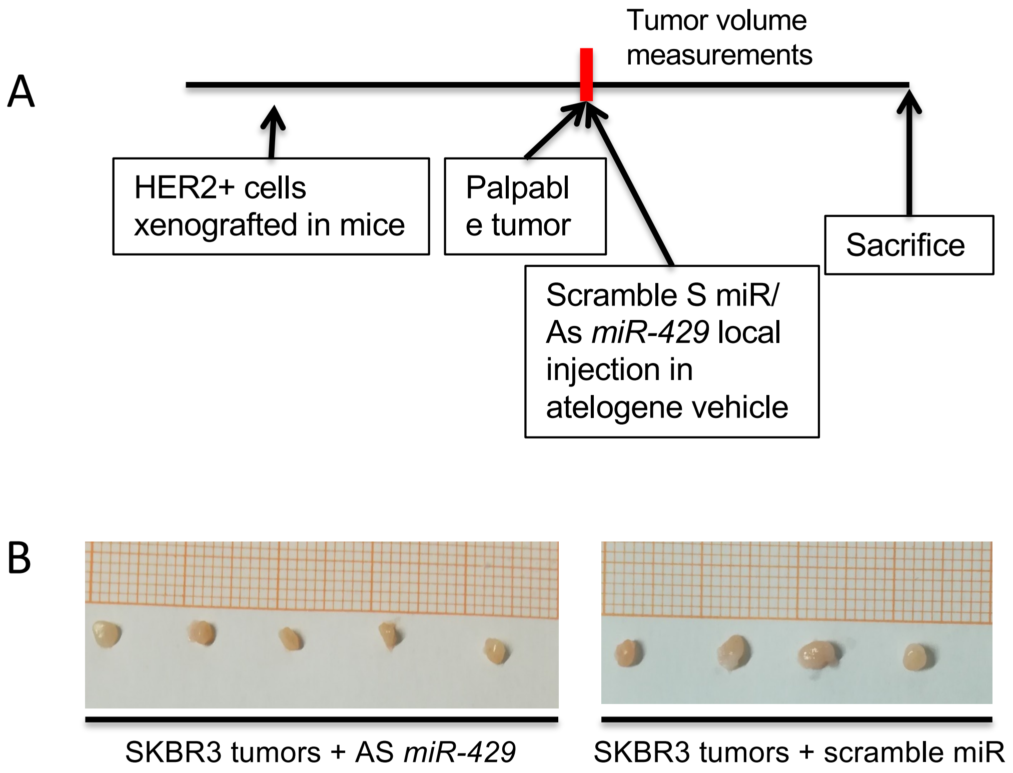

Figure S6 *VHL* is the direct target of *miR-429*.
Sequence analysis of the 3’ UTR of human *VHL* revealed a seed for *miR-429* (TargetScan prediction)(A).

VHL 3’UTR 822-828	…UUUGAUUAU**AGUAUUA**A…
Hsa-miR-429 (MIMAT0001536)	AAUGGUCUG**UCAUAAU**

Figure S7.

RT-PCR analysis on tumors formed by scramble- or As miR-429 treated SKBR3 cells, explanted from mouse. The tumors, in which we succeeded in *miR-429* reduction (A), have a higher VHL expression (B), reduction in HIF1α-target SLUG (C), VEGF (D) and SNAIL (E) expression in respect to Scramble-treated tumors. T test, p value <0.05, *; < 0.01, **.

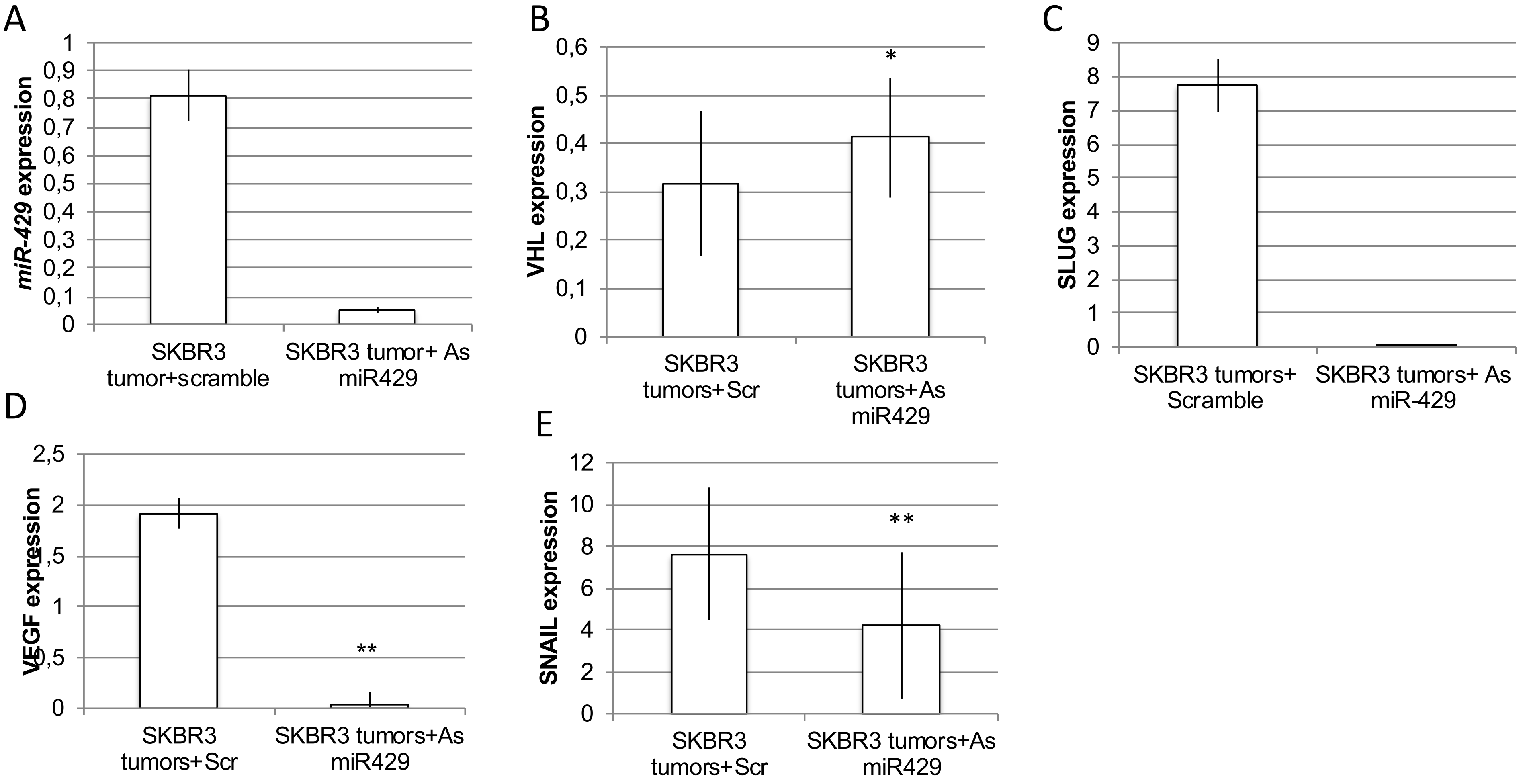

Supplement: Supplementary file 1 — Supplementary figures and tables. [file thnov10p0050s1.pdf]
